# Patterns of venous thromboembolism risk in patients with localized colorectal cancer undergoing adjuvant chemotherapy or active surveillance: an observational cohort study

**DOI:** 10.1186/s12885-017-3392-4

**Published:** 2017-06-15

**Authors:** Jakob Michael Riedl, Florian Posch, Angelika Bezan, Joanna Szkandera, Maria Anna Smolle, Thomas Winder, Christopher H. Rossmann, Renate Schaberl-Moser, Martin Pichler, Michael Stotz, Herbert Stöger, Armin Gerger

**Affiliations:** 1 Division of Clinical Oncology; Department of Medicine; Comprehensive Cancer Center Graz, Medical University of Graz, Auenbruggerplatz 15, 8036 Graz, Austria; 2 Department of internal medicine II, LKH Feldkirch, Carinagasse 47, A-6807 Feldkirch, Austria; 3 Center for Biomarker Research in Medicine, Stiftingtalstrasse 5, 8010 Graz, Austria; 4 Department of experimental therapeutics, The University of Texas MD Anderson Cancer Center, 1901 Eastroad, Houston, TX 77054 USA

**Keywords:** Venous thromboembolism, Thrombosis, Colorectal cancer, Recurrence, Adjuvant chemotherapy

## Abstract

**Background:**

Venous thromoboembolism (VTE) is a frequent and burdensome complication of metastatic colorectal cancer (CRC). However, the epidemiology of VTE in patients with localized CRC after surgery in curative intent is incompletely understood. In this single-center observational cohort study, we investigate patterns of VTE risk in localized CRC, and define its relationship with baseline risk factors, adjuvant chemotherapy and CRC recurrence.

**Methods:**

Five-hundred-sixteen patients with stage II/III CRC were included retrospectively at the time of surgery, and followed until the occurrence of VTE, CRC recurrence, or death (median age = 65.1 years, stage II and III: *n* = 151 (29.5%), *n* = 361 (70.5%); adjCTX: *n* = 339 (65.7%)).

**Results:**

During a median follow-up of 2.7 years, 15 VTEs (2.7%) and 116 recurrences (22.5%) occurred, and 46 patients (8.9%) died. Six-month, 1-year, and 5-year VTE risks were 1.6%, 2.0% and 3.2%, respectively. In competing risk time-to-VTE regression, adjCTX was not associated with an increased risk of VTE (Subdistribution hazard ratio = 0.98, 95% CI:0.33–2.88, *p* = 0.97). The occurrence of disease recurrence strongly increased the risk of VTE (Multi-state model: Transition hazard ratio (THR) = 13.03, 95% CI:4.39–38.74, *p* < 0.0001)). Conversely, the onset of VTE did not predict for recurrence (THR = 1.95, 95% CI: 0.62–6.16, *p* = 0.25).

**Conclusion:**

VTE risk is very low in localized CRC and does not appear to be increased by adjuvant chemotherapy. Thus, primary thromboprophylaxis is unlikely to result in clinical benefit in this population. The strongest determinant of VTE risk appears to be disease recurrence.

## Background

Venous thromboembolism (VTE) is a frequent complication of malignancy and a leading cause of death in patients with cancer [1]. While the risk of VTE varies greatly between different tumor entities, colorectal cancer (CRC) has been described as a high-VTE-risk disease entity [2]. With a pooled incidence of 33 VTE events per 1000 person-years, CRC harbors the second highest risk of VTE among the four most common cancers in the western world [3]. The majority of data on VTE risk in CRC derives from patients with metastatic disease. High tumor burden, antineoplastic therapy, and reduced performance status exacerbate VTE risk in this setting [4–6]. In contrast, patterns of VTE risk in the localized setting of CRC remain ill-defined. Well-established risk factors for VTE, such as surgery, radiotherapy and antineoplastic treatment, are highly prevalent in current neoadjuvant or adjuvant treatment concepts for localized CRC [7, 8]. Understanding the patterns of VTE risk in this patient population may therefore foster the identification of high-VTE-risk-patients who could benefit from primary thromboprophylaxis. A further important epidemiological aspect is that the relationship between VTE, disease recurrence and death has not been conclusively established in localized CRC.

The study aims to define the patterns of VTE risk in localized CRC. The analysis will draw on observational data to estimate the risk of VTE in localized CRC after curative surgery, and define its association with baseline risk factors, adjuvant chemotherapy and disease recurrence.

## Methods

### Study population and design

Adult patients with stage II or III histologically-verified, localized adenocarcinoma of the colon or rectum referred to our Oncology Division between January, 2010 and March, 2015 represented the population of this single-center, retrospective cohort study. All patients with UICC stage IV disease were excluded. Further, patients with neuroendocrine tumors/carcinomas were excluded. However, patients on permanent anticoagulation prior surgery (e.g. for stroke prevention in atrial fibrillation) were eligible for inclusion. Baseline and outcome data were collected retrospectively from our prospectively-maintained electronic healthcare database. The primary endpoint of this study was a composite of objectively-confirmed, symptomatic or incidental deep vein thrombosis and/or pulmonary embolism occurring after surgery in curative intent. VTE events that occurred during neoadjuvant therapy or before tumor diagnosis were not counted as a primary outcome event. Secondary endpoints included disease recurrence and death. Disease recurrence was defined as a composite of local recurrence and/or distant metastasis, whatever came first.

### Statistical methods

**Fig. 1:**
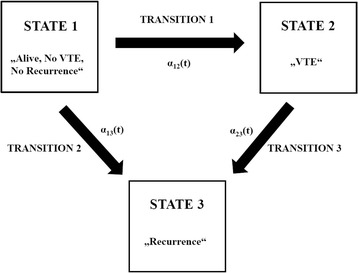
A unidirectional illness-death model for VTE and recurrence in patients with localized CRC after surgery. The transition hazards for the respective transitions between the states are labeled as α_xy_(t), respectively. In this three-state, three-transition unidirectional illness-death model, the states 1, 2, and 3 represent an initial, transient, and absorbing state, respectively. In state#1, patients are alive and free from recurrence and VTE after curative surgery. They can either remain in this “initial” state, transit into the “intermediate” state#2 (transition#1), or transit into the “absorbing” state#3 (“recurrence”) either directly from state#1 (transition#2) or from state#2

All statistical analyses were performed using Stata (Windows version 13.0, Stata Corp., Houston, TX, USA) and R (Windows version 3.1.1., R Core Team (2014), The R Foundation for Statistical Computing, Vienna, Austria). Continuous variables were reported as medians [25th–75th percentile], whereas categorical data were summarized as absolute frequencies and percentages. For comparing means between two or more groups, we used Wilcoxon rank-sum tests and Kruskal-Wallis tests [9, 10]. The association between two categorical variables was assessed with χ^2^-tests or Fisher’s exact tests, as appropriate [11, 12]. Median follow-up was calculated with the inverse Kaplan-Meier estimator according to Schemper & Smith [13]. For the estimation of VTE risk and recurrence risk, we implemented competing risk cumulative incidence estimators according to Marubini & Valsecchi, considering death-from-any-cause as the competing event of interest (Stata routine stcompet) [14]. The 1-Kaplan-Meier estimator was used for calculating the risk of death-from-any-cause [15]. To dissect the temporal associations between recurrence and VTE, VTE and recurrence, and VTE and death, we fitted three unidirectional illness-death models (Schematic representation: Fig. 1). These multi-state analyses were performed in R, using the mstate library [16]. Proportional baseline hazards were specified for transitions #2 and #3 (PH models) [17]. To study the impact of VTE *time point* on mortality, we extended the multi-state models by including the *time-to-VTE* as a covariate for transition#3 (*State arrival extended* (SAE) model). For multi-state based predictions, we generated transition hazards and state occupation probabilities with the msfit and probtrans (implementation of the Aalen estimator) functions of the mstate library [16]. The full analysis code is provided on request from the authors. A general model building framework for multi-state analysis, and relationships with competing risk analysis, are discussed elsewhere [16, 18].

## Results

### Analysis at baseline

Five-hundred-sixteen patients were included in the analysis (Table 1). At baseline, the median age of the cohort was 65.1 years (range 24–91). Approximately half of the cohort suffered from rectal cancer (*n* = 246 (47.9%)), and slightly more than two thirds of patients had stage III disease. Further, two out of three patients were treated with adjuvant chemotherapy (adjCTX) after surgery (*n* = 339 (65.7%)). As compared to patients managed with active surveillance, patients receiving adjCTX were younger (median age: 61.6 vs. 70.4 years, *p* < 0.0001) more likely to have stage III disease (prevalence of stage III: 79.8% vs. 55.7%, *p* < 0.0001) and had a better performance status (prevalence of patients with a Karnofsky Index ≤ 80%: 11.1% vs. 28.9%, *p* < 0.0001).

**Table 1:** Baseline characteristics of the study population

Variable	n (%miss.)	Overall (*n* = 516)	No VTE (*n* = 501)	VTE (*n* = 15)	p*
Age at diagnosis (years)	516 (0.0%)	65.1 [55.3–72.3]	65.2 [55.2–72.4]	62.1 [57.0–67.1]	0.28
BMI (kg/m^2^)	413 (20.0%)	25.5 [23.0–28.7]	25.4 [22.8–28.7]	29.9 [25.6–36.6]	0.005
Karnofsky Index at diagnosis (%)	347 (32.8%)	/	/	/	0.23
≤ 80%	/	57 (16.4%)	56 (16.6%)	1 (11.1%)	/
90%	/	116 (33.4%)	115 (34.0%)	1 (11.1%)	/
100%	/	174 (50.1%)	167 (49.4%)	7 (77.8%)	/
Family history of CRC	256 (50.4%)	/	/	/	0.76
No family history	/	217 (84.8%)	211 (84.7%)	6 (85.7%)	/
1^st^ degree relative	/	25 (9.8%)	24 (9.6%)	1 (14.3%)	/
2^nd^ degree relative	/	14 (5.5%)	14 (5.6%)	0 (0.0%)	/
Smoker or Ex-Smoker	358 (30.6%)	121 (33.8%)	117 (33.5%)	4 (44.4%)	0.49
Tumor localization	514 (0.4%)	/	/	/	0.84
Cecum/Appendix	/	56 (10.9%)	56 (11.2%)	0 (0.0%)	/
Ascending colon	/	37 (7.2%)	35 (7.0%)	2 (13.3%)	/
Right flexure	/	15 (2.9%)	14 (2.8%)	1 (6.7%)	/
Transverse colon	/	19 (3.7%)	18 (3.6%)	1 (6.7%)	/
Left flexure	/	22 (4.3%)	21 (4.2%)	1 (6.7%)	/
Descending colon	/	9 (1.75%)	9 (1.8%)	0 (0.0%)	/
Sigma	/	107 (20.8%)	104 (20.8%)	3 (20.0%)	/
Rectum	/	246 (47.9%)	239 (47.9%)	7 (46.7%)	/
≥ 2 localizations	/	3 (0.6%)	3 (0.6%)	0 (0.0%)	/
TNM	509 (1.4%)	/	/	/	0.75
T1	/	9 (1.8%)	9 (1.8%)	0 (0.0%)	/
T2	/	27 (5.3%)	27 (5.5%)	0 (0.0%)	/
T3	/	372 (73.1%)	360 (72.9%)	12 (80.0%)	/
T4	/	101 (19.8%)	98 (19.8%)	3 (20.0%)	/
TNM	503 (2.5%)	/	/	/	0.39
N0	/	149 (29.6%)	144 (29.5%)	5 (33.3%)	/
N1	/	215 (42.7%)	211 (43.2%)	4 (26.7%)	/
N2	/	139 (27.6%)	133 (27.3%)	6 (40.0%)	/
Stage	512 (0.8%)	/	/	/	0.81
Stage II	/	151 (29.5%)	147 (29.6%)	4 (26.7%)	/
Stage III	/	361 (70.5%)	350 (70.4%)	11 (73.3%)	/
Adjuvant chemotherapy	516 (0.0%)	339 (65.7%)	329 (65.7%)	10 (66.7%)	0.94

Distribution overall and by total recurrence status. Continuous variables are summarized as medians [25th percentile (Q1) – 75th percentile (Q3)], whereas categorical variables are reported as absolute frequencies and percentages. *p-values for difference between non-VTE and VTE group are from Pearson’s chi-squared tests (categorical variables with expected cell counts ≥5), Fisher’s exact tests (categorical variables with expected cell counts <5), or Wilcoxon rank-sum tests (continuous variables)

*Abbreviations*: *KI* – Karnofsky Index, *TNM* – Tumor Node Metastasis classification, *BMI* – Body mass index, *CRC* – Colorectal cancer

### Analysis of event rates

After a median follow-up interval of 2.7 years (range: 18 days – 5.0 years), 15 patients (2.7%) developed VTE, 116 patients (22.5%) developed recurrence, and 46 patients (8.9%) died. Among the 15 VTE events, we observed 10 (66.6%) DVTs, 4 (26.7%) PEs, and 1 patient (6.7%) developed both DVT and PE at the same time. Five venous thrombotic occurrences were not counted as events: Recurrent PE after first in-study PE (*n* = 1), Subclavian vein thrombosis associated with a port-a-cath device (*n* = 1), portal vein thrombosis (*n* = 2), DVT during induction chemotherapy after an insufficient response to neoadjuvant chemoradiation (*n* = 1). Among the 116 recurrences, 13 (11.2%) were local recurrences, 98 (84.5%) were distant metastasis, and 5 patients (4.3%) suffered from concurrently detected local recurrence and distant metastasis. In competing risk analysis, the cumulative 6-month, 1-year, and 5-year incidence of VTE was 1.6% (95% CI: 0.7–3.0), 2.0% (1.0–3.5), and 3.2% (1.9–5.1), respectively. The corresponding risks of recurrence were 5.7% (3.9–8.0), 13.1% (10.3–16.3), and 28.6% (23.6–33.8), respectively.

### Predictors of VTE

**Fig. 2:**
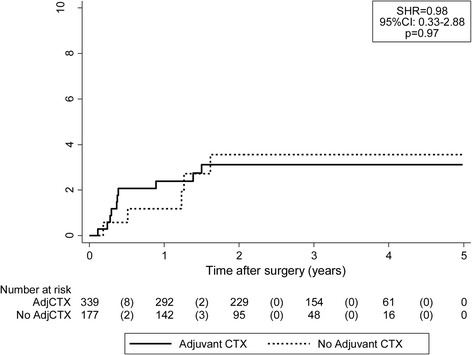
Cumulative incidence of VTE according to adjuvant chemotherapy status. Note that the y-axis scaling only continues until 10%

Among the variables reported at baseline, only BMI did significantly differ between patients that did and did not develop VTE during follow-up, with a higher baseline BMI reported in patients developing VTE (*p* = 0.005, Table 1). In univariable time-to-VTE competing risk regression (Table 2), a higher BMI emerged as a significant predictor of an increased risk of VTE (Subdistribution hazard ratio (SHR) per 5 kg/m^2^ increase in BMI = 1.57, 95% CI: 1.23–2.02, *p* < 0.0001). Importantly, adjCTX was not associated with a higher risk of VTE (SHR = 0.98, 95% CI: 0.33–2.88, *p* = 0.97). In detail, the 6-month, 1-year, and 5-year risks of VTE were 2.1%, 2.4% and 3.1% in the adjCTX group, and 0.6%, 1.2% and 3.6% in the active surveillance group (Gray’s test p=, Fig. 2). As the clinical profile of patients with adjCTX significantly differed from patients undergoing active surveillance, we performed an inverse probability of treatment waited (IPTW) analysis including the variables age, BMI, stage, T of TNM, N of TNM, Karnofsky index and smoking status. Also here, we did not observe an association between adjCTX and venous thromboembolic events (waited SHR = 0.47, 95% CI: 0.09–2.34, *p* = 0.35).

**Table 2:** Uni- and multivariable predictors of VTE risk in localized CRC

	Univariable analysis			Multivariable analysis adjusting for BMI		
Variable	Subdistribution hazard ratio (SHR)	95% CI	*p*	Subdistribution hazard ratio (SHR)	95% CI	*p*
Age at diagnosis (per 5 years increase)	0.93	0.81–1.06	0.29	0.85	0.70–1.02	0.09
BMI (per 5 kg/m^2^ increase)	1.57	1.23–2.02	<0.0001	N/A	N/A	N/A
KI at diagnosis						
100%	Ref.	Ref.	Ref.	Ref.	Ref.	Ref.
< 100%	0.28	0.06–1.35	0.11	0.31	0.07–1.48	0.14
Family history of CRC	1.00	0.12–8.23	0.99	1.19	0.14–10.22	0.87
Smoker or Ex-Smoker	1.64	0.44–6.09	0.46	1.71	0.43–6.85	0.45
Tumor localization						
Non-rectal	Ref.	Ref.	Ref.	Ref.	Ref.	Ref.
Rectal	0.97	0.35–2.68	0.95	1.74	0.5–6.11	0.39
TNM – T						
T1 & T2 & T3	Ref.	Ref.	Ref.	Ref.	Ref.	Ref.
T4	0.95	0.27–3.36	0.94	0.53	0.06–4.80	0.57
TNM - N						
N0	Ref.	Ref.	Ref.	Ref.	Ref.	Ref.
N1 & N2	0.85	0.29–2.46	0.76	1.14	0.28–4.66	0.86
Stage						
Stage II	Ref.	Ref.	Ref.	Ref.	Ref.	Ref.
Stage III	1.15	0.37–3.59	0.81	1.75	0.40–7.63	0.46
Adjuvant chemotherapy	0.98	0.33–2.88	0.97	1.18	0.27–5.24	0.23

*Abbreviations*: *SHR* Subdistribution hazard ratio, *95% CI* 95% confidence interval, *KI* Karnofsky Index, *TNM* Tumor Node Metastasis classification, *BMI* Body mass index, *CRC* Colorectal cancer

### Relationship between VTE, recurrence, and death

In contingency analysis, VTE and recurrence were highly associated with each other (Chi-Squared *p* < 0.001). In multistate analysis, the onset of recurrence was associated with a 13-fold increase in the risk of VTE (THR = 13.03, 95% CI: 4.39–38.74, *p* < 0.0001). This finding prevailed after adjusting for BMI (THR = 12.36, 95% CI: 3.32–46.06, *p* < 0.001). In contrast, we did not observe an association between VTE occurrence and a higher risk of cancer recurrence (THR = 1.95, 95% CI: 0.62–6.16, *p* = 0.25). Recurrences lead to an 18-fold increase in the risk of death (transition hazard ratio (THR) = 18.37, 95% CI = 9.12–37.00, *p* < 0.0001), whereas the onset of VTE was only a weak predictor of an increased risk of death (THR = 2.76, CI = 0.85–8.95, *p* = 0.09).

## Discussion

In this study, we aimed to define patterns of VTE risk in patients with localized colorectal cancer after curative surgery. Overall, we found a very low risk of VTE in the total cohort as well as in the patients who underwent adjuvant chemotherapy. Importantly, adjuvant chemotherapy did not emerge to be a predictor of an increased risk of VTE in this cohort, which does not support the concept that patients undergoing adjuvant chemotherapy after curative surgery for colorectal cancer benefit from primary thromboprophylaxis. The strongest determinant of VTE risk was disease recurrence. (Clinical practis points summarized in Table 3).

**Table 3:** Clinical practice points of this study

• In contrast to metastatic colorectal cancer, patients with localized colorectal cancer have a very low risk of VTE during the first three years after surgery in curative intent.
• Adjuvant chemotherapy does not appear to increase the risk of VTE.
• Primary thromboprophylaxis beyond the recommended extended post-surgical thromboprophylaxis period of 28 days is unlikely to provide clinical benefit in this population.
• Disease recurrence after localized colorectal cancer increases the risk of VTE by more than 10-fold.

Cancer is a major risk factor for VTE [19–21]. Although the risk of cancer-associated VTE strongly varies between tumor types, metastatic CRC is considered to be a high-VTE-risk tumor entity, with up to 15% of patients developing VTE during their course of illness [22]. The risk of VTE in patients with metastatic CRC is highest during the first 6 months after diagnosis, and appears to be further increased by chemotherapy [23–25]. Whether patients with mCRC would benefit from primary thromboprophylaxis is therefore an important area of ongoing research [26]. In the present study including patients with localized CRC, we observed a very different pattern of VTE risk. First, we observed that the overall risk of VTE for up to 5 years after surgery was as low as 3.2%. Around half of the 15 VTE events observed in this study occurred during the first six months after surgery. This suggests that the post-surgical period is a contributor to VTE risk in CRC patients, and that adherence to extended thromboprophylaxis guidelines in our cohort was high. Interestingly, adjuvant chemotherapy did not emerge to be associated with a higher risk of VTE. This is in contrast to the metastatic setting, where it is believed that chemotherapy is a major contributor towards thrombotic risk. However, chemotherapy is likely not a causal risk factor for cancer-associated VTE, but rather leads to a liberation of prothrombotic agents from cancer cells, including tissue factor bearing microparticles, cell-free DNA, and histones [27, 28].

The low risk of VTE in our cohort of patients with localized disease and the lack of association with adjuvant chemotherapy does not support the hypothesis that patients undergoing adjuvant chemotherapy would benefit from primary thromboprophylaxis for the duration of cytotoxic chemotherapy. Assuming a 50% relative reduction in the risk of VTE with primary thromboprophylaxis during a 6-month course of adjuvant chemotherapy, this intervention would - according to our 6-month VTE risk of 2.1% in the adjuvant therapy group – result in a number needed to treat of 125 [29]. Treating 125 patients with a low molecular weight heparin prophylaxis for 6-months to prevent one VTE event would not only incur significant healthcare costs, but also set an excessive number of patients at risk for bleeding complications [30, 31]. Thus, the main message of this study is that primary thromboprophylaxis should not be given to patients with localized CRC after surgery *beyond* the recommend 28 days of extended post-surgical prophylaxis.

Several predictors for an increased risk of VTE have been identified in patients with metastatic cancers, including – among others - tumor type, lymph node metastasis, elevated D-Dimer, elevated levels of soluble p-selectin, elevated BMI, elevated leukocytes and platelet counts, and anemia/use of erythropoiesis-stimulating agents (ESAs) [32–38]. However, in this study on patients with localized CRC, we could only identify an elevated BMI as an independent predictor of a higher venous thromboembolic risk. A borderline association was observed between advanced age at surgery and a lower risk of VTE. We can speculate that this may be explained by a confounding influence of a more aggressive malignancy in younger patients. In summary, this supports the concept that risk factors for VTE in localized CRC and metastatic CRC may be quite different, and VTE risk prediction tools for the metastatic setting, such as the Khorana score, may not necessarily generalize to patients with localized CRC [36]. Rather, our study suggests that risk factors for VTE in the adjuvant setting of localized CRC are more similar to VTE risk factors in the general population. This notion is particularly supported by the observed strong association of an elevated BMI with VTE in our study.

The temporal relationship between individual endpoints, such as VTE, recurrence, and death can be complex. We therefore implemented multi-state models that allowed us to statistically dissect how VTE and recurrence associate with each other over time, and how they influence the risk of death. Here, we observed that recurrence was a very strong contributor towards VTE risk. The onset of recurrence increased the risk of developing VTE by a factor of more than 13. This excessive risk increase reflects the transition of patients from the low-VTE-risk localized setting into the high-VTE- recurrent/metastatic setting, and indicates that recurrence is a much stronger risk factor for VTE in localized CRC than any of the baseline variables assessed in this study. Conversely, we only observed a weak and statistically non-significant trend for a relationship between the occurrence of VTE and a higher risk of recurrence. In patients without any evidence of cancer, an “unprovoked” VTE can be an early sign of malignancy [39]. In our study, the absence of a strong association between VTE occurrence and cancer recurrence does not suggest that VTE occurrence may be an early clinical indicator of cancer recurrence. Therefore, we cannot recommend that a VTE event in such a patient should per se lead to a full diagnostic work-up for cancer recurrence. As expected, recurrence emerged as a strong risk factor for death.

Finally, we want to discuss some limitations of this study. First, its retrospective cohort design with retrospective retrieval of thrombotic event data from our in-house electronic healthcare database may have led to an underreporting of VTE events, and thus an underestimation of VTE risk in this study. Further, data on anticoagulation at baseline were not systematically collected, which could have resulted in a “dilution” of VTE risk over time by patients that received continuous anticoagulation for reasons such as atrial fibrillation. Third, within our geographical region, only a fraction of patients suffering from stage I colorectal cancer are referred to our Division of Clinical Oncology. Therefore, we had to exclude stage I patients from this analysis to minimize potential selection bias. However, all patients with stage II and III disease are routinely referred to our Division in our region, wherefore our data are generalizable to these patient populations. Fourth, VTE risk may be higher in patients undergoing neoadjuvant chemoradiation for rectal cancer. However, our study does not systematically investigate this treatment period, but rather analyzes VTE risk patterns after surgery. A fifth limitation of our study is that validated biomarkers for cancer-associated VTE in the metastatic CRC setting, such as D-Dimer, were not measured in this study. Moreover, due to the retrospective nature of data ascertainment, we were not able to collect potentially important data on thrombophilia and past history of VTE. This limitation also applies to data on permanent anticoagulation, which were not systematically collected. Permanent anticoagulation, for example to prevent stroke in patients with atrial fibrillation, may have lead to an underestimation of VTE risk in our study. However, given the expected low prevalence of atrial fibrillation in a population with a median age of 65.1 years, we carefully speculate that the magnitude of such a bias will likely be small. Finally, we want to mention two strengths of this study, namely the rigorous adjudication of VTE events, and the application of a statistical methodology (“multi state”) that can dissect the complex temporality between VTE, recurrence, and death.

## Conclusion

In this study with a median follow-up of almost 3 years, we have shown that the risk of VTE in patients with localized CRC after curative surgery is very low. Importantly, adjuvant chemotherapy did not emerge as a risk factor for VTE in this setting. Consequently, primary thromboprophylaxis beyond the recommended extended post-surgical thromboprophylaxis is unlikely to provide clinical benefit. The strongest determinant for VTE in this setting is disease recurrence.

## Abbreviations

adj CTX: Adjuvant chemotherapy
BMI: Body mass index
CI: Confidence interval
DVT: Deep vein thrombosis
ESAs: Erythropoiesis-stimulating agents
mCRC: Metastatic colorectal cancer
PE: Pulmonary embolism
SHR: Subdistribution hazard ratio
THR: Transition hazard ratio
VTE: Venous thromboembolism
